# Comparative morphology of the axial complex and interdependence of internal organ systems in sea urchins (Echinodermata: Echinoidea)

**DOI:** 10.1186/1742-9994-6-10

**Published:** 2009-06-09

**Authors:** Alexander Ziegler, Cornelius Faber, Thomas Bartolomaeus

**Affiliations:** 1 Institut für Immungenetik, Charité-Universitätsmedizin Berlin, Freie Universität Berlin, Thielallee 73, 14195 Berlin, Germany; 2 Institut für Klinische Radiologie, Universitätsklinikum Münster, Westfälische Wilhelms-Universität Münster, Waldeyerstraße 1, 48149 Münster, Germany; 3 Institut für Evolutionsbiologie und Zooökologie, Rheinische Friedrich-Wilhelms-Universität Bonn, An der Immenburg 1, 53121 Bonn, Germany

## Abstract

**Background:**

The axial complex of echinoderms (Echinodermata) is composed of various primary and secondary body cavities that interact with each other. In sea urchins (Echinoidea), structural differences of the axial complex in "regular" and irregular species have been observed, but the reasons underlying these differences are not fully understood. In addition, a better knowledge of axial complex diversity could not only be useful for phylogenetic inferences, but improve also an understanding of the function of this enigmatic structure.

**Results:**

We therefore analyzed numerous species of almost all sea urchin orders by magnetic resonance imaging, dissection, histology, and transmission electron microscopy and compared the results with findings from published studies spanning almost two centuries. These combined analyses demonstrate that the axial complex is present in all sea urchin orders and has remained structurally conserved for a long time, at least in the "regular" species. Within the Irregularia, a considerable morphological variation of the axial complex can be observed with gradual changes in topography, size, and internal architecture. These modifications are related to the growing size of the gastric caecum as well as to the rearrangement of the morphology of the digestive tract as a whole.

**Conclusion:**

The structurally most divergent axial complex can be observed in the highly derived Atelostomata in which the reorganization of the digestive tract is most pronounced. Our findings demonstrate a structural interdependence of various internal organs, including digestive tract, mesenteries, and the axial complex.

## Background

"Das Dorsalorgan ist von jeher das Schmerzenskind der Anatomen gewesen." (Johannes Wagner, 1903)

All echinoderms (Echinodermata) possess an axial complex as part of their coelomic and haemal system. This organ complex is characterized by a structural, functional and topographic interaction between various primary and secondary body cavities and is composed of derivatives of the three paired larval coeloms, i.e. the protocoel and the mesocoel, both surrounded by the lining epithelia of the metacoel. The different coelothelia rest on the connective tissue matrix that is crossed by numerous haemal spaces and lacunae. Primary and secondary body cavities can be distinguished at the ultrastructural level by their lining [1-3]: a primary body cavity is lined by extracellular matrix (ECM), whereas a secondary body cavity is lined by an epithelium consisting of basal lamina and epithelial cells. In echinoderms, the primary body cavities form the haemal system, whereas the secondary body cavities develop to constitute the coelom and are thus also termed coelomic cavities. Historically, the secondary body cavities found in echinoderms have been termed axocoel (protocoel), hydrocoel (mesocoel), and somatocoel (metacoel) [4].

The axial complex of sea urchins (Echinoidea) is part of this tripartite coelomic system. In its most basic form, the axial complex lies vertically within the oral-aboral axis – hence the term "axial complex" – of interradius CD (interambulacrum 2) and is surrounded by the oral and aboral somatocoel. It consists of derivatives of axocoel and hydrocoel. During ontogenesis, these two cavities have different fates: while the right hydrocoel degenerates, the left hydrocoel gains connection to the left axocoel via the stone canal. During further ontogenesis, the left hydrocoel becomes the ring canal that gives rise to the radial canals of the ambulacral (or water vascular) system, and the stone canal connects the ring canal with the madreporic ampulla. The latter is connected to the exterior by a number of small ductules, the madreporic pore canals. These canals penetrate the madreporic plate, their distal regions being lined by an ectodermally derived epithelium, the epidermis. As in sea stars (Asteroidea), the lining of the proximal madreporic pore canal sections is of mesodermal origin [5], since madreporic ampulla and axial coelom ontogenetically originate from the left larval axocoel.

**Figure 1:**
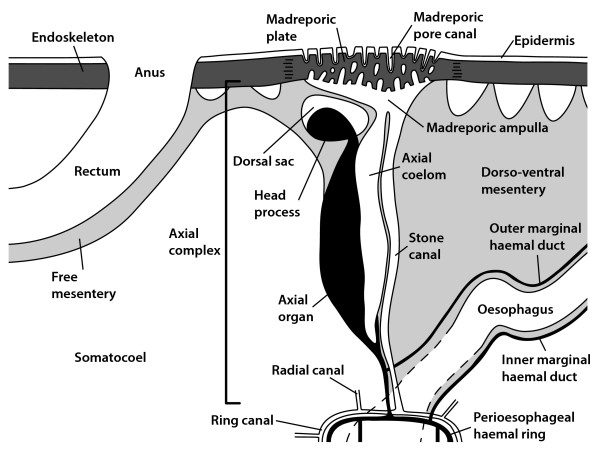
**Semi-schematic representation of the echinoid axial complex**. Semi-schematic representation of the apical region in interradius CD (interambulacrum 2) of *Sphaerechinus granularis *(Echinoidea: Echinoida) showing madreporic plate, ring canal, axial complex, and rectum [after Leipoldt [26] and Strenger [61], modified]. Aboral haemal ring, Aristotle's lantern, gonads, gonoducts, and spongy (or Tiedemann's) bodies not shown. Not to scale.

The axial coelom is an orally oriented part of the axocoel that partly enwraps the stone canal as well as the axial organ (see Fig. 1 for a representative example). The axial organ is a large space within the connective tissue matrix, lined by epithelial cells of the coelomic cavities that surround it. It constitutes in fact a hypertrophy of the mesenteries that attach part of the digestive tract to the calcite endoskeleton. Since the haemal structures of the axial complex are mainly located within the dorso-ventral mesentery, bounded by the lining of the somatocoel, the axial organ is surrounded by the somatocoel on one side and by the axocoel on the other. The axial organ is an integral component of the echinoid haemal system [6]. An aboral extension of the axial organ, however, is surrounded by the so-called dorsal sac, a derivative of the right larval axocoel. This aboral extension is termed the head process and consists of a large compartment within the connective tissue matrix between somatocoel and dorsal sac. Axial organ and head process are crossed by numerous anastomosing haemal lacunae that aborally join with the anal haemal ring and the genital lacunae. In addition, the axial organ is crossed by numerous canaliculi that constitute invaginations of the axial coelom and somatocoelomic epithelia. Adorally, the axial organ does not end entirely blindly, but extends into the perioesophageal haemal ring either directly or through connecting haemal lacunae.

**Figure 2:**
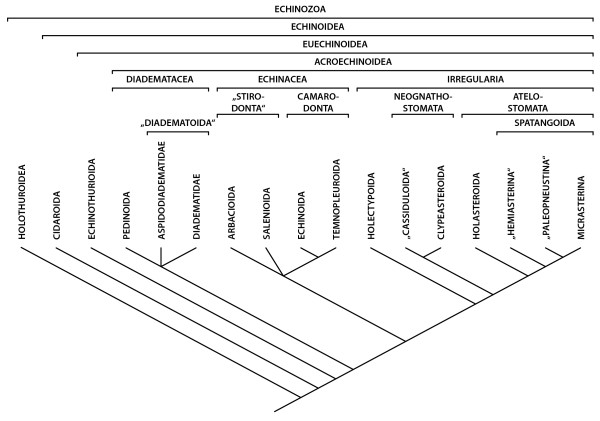
**Current understanding of sea urchin phylogeny**. The hypotheses of echinozoan and echinoid relationships are based on multiple sources of morphological and molecular datasets (for further references see the Materials and methods section). This tree has not been generated using a consensus or numerical technique and reflects the views and biases of the authors.

The various sub-structures forming the axial complex have been successfully homologized in all echinoderm taxa [7-13]. In sea urchins – see Fig. 2 for the present view on sea urchin phylogeny based largely on hard-part morphology and molecular data – the morphological data obtained for this structure are based to a large extent on findings in the more easily accessible "regular" sea urchins such as *Arbacia punctulata*, *Psammechinus miliaris*, *Sphaerechinus granularis*, and *Strongylocentrotus purpuratus*. However, historical [14-18], as well as more recent [19,20] studies have revealed that the axial complex found in irregular sea urchins differs in its gross morphology and histology from that found in "regular" taxa. This is exemplified by the work of Kaburek and Hilgers [20], who reported that *Schizaster canaliferus *possesses a specialized axial complex exhibiting pronounced structural changes including loss of sub-structures. However, some descriptions of sea urchin soft tissues [21-24] indicate that there is good reason to believe that the axial complex found in *Schizaster canaliferus *might not be typical for all Irregularia. In the more primitive irregular species *Echinoneus cyclostomus*, for example, the gross morphology of the axial complex is more similar to the "regular" type. A number of questions therefore arise from previous studies: (i) What are the major changes affecting the architecture of the axial complex within the Echinoidea? (ii) Can significant differences in its structure be observed also among the "regular" species? (iii) What could have caused the drastic changes found in some taxa? (iv) Does a better understanding of axial complex morphology reveal information about its function? And finally, (v) can characters be deduced from comparative observations of the axial complex that might be useful for phylogenetic inferences?

Using non-invasive imaging techniques that permit large taxon sampling [24,25], we analyzed numerous species from almost all sea urchin orders to infer the structure of their axial complex. These data were extended employing invasive techniques such as dissection, histology, as well as transmission electron microscopy, and included also findings from published studies spanning almost two centuries. The combined analysis suggests an interdependence of soft tissue organ systems in sea urchins that we believe to be ultimately responsible for the structural changes of the axial complex observed in irregular taxa.

Since part of the confusion regarding axial complex morphology can be attributed to the bewildering terminology applied by different authors and in different languages to the same anatomical entities, we also suggest a list of definitions and provide a multilingual compilation for echinoid axial complex components to facilitate further comparative studies.

## Materials and methods

The specimens referred to in this study are listed in Tables 1 &2 together with information on the current systematic classification of each species, the source of the data used in the study, specimen ID where applicable, and literature references [26-67].

**Table 1:** List of "regular" sea urchin species included in this study.

**Order**	**Family**	**Species**	**Method used**	**Specimen ID**	**Reference**
Cidaroida Claus, 1880	Histocidaridae Lambert, 1900	*Histocidaris elegans* (Agassiz, 1879)	MRI (81 μm)^3^	ZMH E907	this study


	Cidaridae Gray, 1825	*Cidaris cidaris *(Linnaeus, 1758)	MRI (81 μm)^3^, dissection, histology	NHM 1925.10.30.103-113	[15,17,26], this study

		*Eucidaris metularia *(Lamarck, 1816)	MRI (81 μm)^3^	NHM 1969.5.1.15-40	[24], this study

		*Eucidaris *sp.	Histology, ultrastructure	-	[27,28]

		*Eucidaris tribuloides *Desmoulins, 1835	Dissection, histology	-	[29], this study

Echinothurioida Claus, 1880	Phormosomatidae Mortensen, 1934	*Phormosoma bursarium *Agassiz, 1881	Dissection, histology	-	[30]

	Echinothuriidae Wyville Thomson, 1872	*Asthenosoma varium* Grube, 1868	Dissection, histology	-	[30,31]

		*Hygrosoma hoplacantha *(Wyville Thomson, 1877)	Dissection	-	[30]

		*Sperosoma biseriatum *Döderlein, 1901	Dissection	-	[30]

Pedinoida Mortensen, 1939	Pedinidae Pomel, 1883	*Caenopedina mirabilis *(Döderlein, 1885)	MRI (81 μm)^3^, dissection	USNM 31178, USNM 31182	this study

Diadematoida Duncan, 1889	Aspidodiadematidae Duncan, 1889	*Aspidodiadema hawaiiense *Mortensen, 1939	MRI (81 μm)^3^, dissection	USNM 27590	this study

		*Plesiodiadema indicum *(Döderlein, 1901)	MRI (81 μm)^3^	ZMB 7232	this study

	Diadematidae Gray, 1855	*Diadema antillarum *Philippi, 1845	Histology	-	[32]

		*Diadema savignyi *Michelin, 1845	MRI (40 μm)^3^	-	this study

		*Diadema setosum *(Leske, 1778)	Dissection, histology	-	this study

Salenioida Delage & Herouard, 1903	Saleniidae Agassiz, 1838	*Salenocidaris hastigera* (Agassiz, 1869)	MRI (81 μm)^3^	ZMB 5816	[24], this study

Arbacioida Gregory, 1900	Arbaciidae Gray, 1855	*Arbacia lixula *(Linnaeus, 1758)	MRI (81 μm)^3^, histology	-	[16,24,32,33], this study

		*Arbacia punctulata *(Lamarck, 1816)	Dissection, histology	-	[34-39]

*Incerta sedis*	Stomopneustidae Mortensen, 1903	*Stomopneustes variolaris *(Lamarck, 1816)	MRI (81 μm)^3^	USNM E45930	[24], this study

Echinoida Troschel, 1872	Parechinidae Mortensen, 1903	*Paracentrotus lividus* (Lamarck, 1816)	MRI (81 μm)^3^, dissection, histology	-	[17,32,33,38,40,41], this study

		*Psammechinus microtuberculatus *(Blainville, 1825)	Dissection, histology	-	[38,42,43]

		*Psammechinus miliaris* (Müller, 1771)	MRI (44 μm)^3^, dissection, histology, ultrastructure	-	[44-47], this study

	Echinidae Gray, 1825	*Echinus esculentus *Linnaeus, 1758	MRI (81 μm)^3^, dissection, histology	ZMB 3826	[18,46,48-52], this study

		*Echinus melo *Lamarck, 1816	Dissection	-	[33]

		*Gracilechinus acutus* (Lamarck, 1816)	Dissection, histology	-	[16,17]

	Echinometridae Gray, 1855	*Echinometra mathaei *(Blainville, 1825)	MRI (81 μm)^3^	NHM 1969.5.1.61-75	this study

		*Echinometra *sp.	Histology, ultrastructure	-	[27]

		*Evechinus chloroticus *(Valenciennes, 1846)	Dissection, histology	-	[53]

	Strongylocentrotidae Gregory, 1900	*Strongylocentrotus dröbachiensis *(Müller, 1776)	Dissection, histology, ultrastructure	-	[7,35,39,54,55]

		*Strongylocentrotus purpuratus *(Stimpson, 1857)	MRI (44 μm)^3^, dissection, histology, ultrastructure	CAS 5724	[24,39,47,54-58], this study

	Toxopneustidae Troschel, 1872	*Lytechinus variegatus *(Lamarck, 1816)	MRI (81 μm)^3^	-	this study

		*Sphaerechinus granularis *(Lamarck, 1816)	MRI (81 μm)^3^, dissection, histology, ultrastructure	-	[15,26,33,59-64], this study

Temnopleuroida Mortensen, 1942	Trigonocidaridae Mortensen, 1903	*Genocidaris maculata* Agassiz, 1869	MRI (36 μm)^3^	ZMB 5827	this study

		*Trigonocidaris albida* Agassiz, 1869	MRI (32 μm)^3^	ZSM 20012468	this study

	Temnopleuridae Agassiz, 1872	*Mespilia globulus *(Linnaeus, 1758)	MRI (44 μm)^3^	ZMB 5620	[24], this study

		*Salmacis bicolor *(Agassiz, 1846)	Dissection, histology	-	[65]

The table provides information on every species studied so far with regard to the axial complex, the method(s) used to infer axial complex anatomy, the specimen ID of museum specimens where applicable, and the respective references. Numbers in brackets behind "MRI" represent the resolution of the dataset. An overview of scanning parameters is provided by [24].

**Table 2:** List of irregular sea urchin species included in this study.

**Order**	**Family**	**Species**	**Method used**	**Specimen ID**	**Reference**
Holectypoida Duncan, 1889	Echinoneidae Agassiz & Desor, 1847	*Echinoneus cyclostomus* Leske, 1778	MRI (86 μm)^3^, dissection	NHM 1969.5.1.105	[22,24], this study

"Cassiduloida" Agassiz & Desor, 1847	Cassidulidae Agassiz & Desor, 1847	*Cassidulus caribaearum *Lamarck, 1801	MRI (81 μm)^3^	CAS 112632	this study

	Echinolampadidae Gray, 1851	*Echinolampas depressa* Gray, 1851	MRI (81 μm)^3^	USNM E32955	[24], this study

	Apatopygidae Kier, 1962	*Apatopygus recens *(Mortensen, 1948)	Dissection	-	[23]

Clypeasteroida Agassiz, 1835	Clypeasteridae Agassiz, 1835	*Clypeaster rosaceus *(Linnaeus, 1758)	MRI (81 μm)^3^	ZMB 2520	this study

	Arachnoididae Duncan, 1889	*Arachnoides placenta *(Linnaeus, 1758)	MRI (81 μm)^3^	ZMB 1439	this study

	Fibulariidae Duncan, 1889	*Echinocyamus pusillus* (Müller, 1776)	MRI 20 × 18 × 18 μm^3^, histology	-	[24,66], this study

	Laganidae Agassiz, 1873	*Peronella lesueuri *Agassiz, 1841	Dissection	MNHN EcEh79	this study

		*Peronella orbicularis* Leske, 1778	Dissection	-	[66]

	Echinarachniidae Lambert, 1914	*Echinarachnius parma* (Lamarck, 1816)	MRI (44 μm)^3^, dissection	ZSM 20011676	[34], Mooi (unpublished data), this study

	Mellitidae Stephanini, 1914	*Mellita quinquesperforata *(Leske, 1778)	Dissection	-	this study

	Astriclypeidae Stephanini, 1911	*Echinodiscus bisperforatus *Leske, 1778	Histology	-	[66]

Holasteroida Durham & Melville, 1957	Urechinidae Duncan, 1889	*Antrechinus nordenskjöldi *(Mortensen, 1905)	Dissection	ZMH E7350	this study

		*Urechinus naresianus *Agassiz, 1879	Dissection	NHM 1903.8.1.100-104	[21], this study

	Pourtalesiidae Agassiz, 1881	*Echinosigra phiale *Mortensen, 1905	Dissection	-	[21]

		*Pourtalesia hispida* Agassiz, 1879	Dissection	ZMH E7349	this study

		*Pourtalesia jeffreysi* Wyville Thomson, 1873	Dissection	-	[21]

		*Pourtalesia wandeli *Mortensen, 1905	MRI (86 μm)^3^, dissection	NHM 1976.7.30.76-95	[21], this study

	Plexechinidae Mooi & David, 1996	*Plexechinus aoetanus (McKnight, 1974)*	Dissection	ZMH E7345	this study

Spatangoida Agassiz, 1840	Hemiasteridae Clark, 1917 ("Hemiasterina")	*Hemiaster expergitus* *(Loven, 1874)*	Dissection	NHM 1914.1.30.66-9	this study

	Schizasteridae Lambert, 1905 ("Paleopneustina")	*Abatus cavernosus *(Philippi, 1845)	MRI (81 μm)^3^	ZMB 5854	[24], this study

		*Schizaster canaliferus *(Lamarck, 1816)	Dissection, SEM, histology	-	[15,20]

	*Incerta sedis* (Micrasterina)	*Heterobrissus niasicus *(Döderlein, 1901)	Dissection, histology	-	[18]

	Spatangidae Gray, 1825 (Micrasterina)	*Spatangus purpureus* Müller, 1776	Dissection, histology	-	[14-17], this study

	Brissidae Gray, 1855 (Micrasterina)	*Brissus unicolor *(Leske, 1778)	Dissection, histology	-	[15,16]

		*Meoma ventricosa *(Lamarck, 1816)	Dissection	-	[8,67]

	Brissopsidae Lambert, 1905 (Micrasterina)	*Brissopsis lyrifera *(Forbes, 1841)	Dissection	-	[15], this study

	Echinocardiidae Wythe Cooke, 1942 (Micrasterina)	*Echinocardium cordatum *(Pennant, 1777)	Dissection, histology, ultrastructure	-	[19], this study

		*Echinocardium flavescens* (Müller, 1776)	Dissection	-	[15]

		*Echinocardium mediterraneum *(Forbes, 1844)	Dissection, histology	-	[16]

The table provides information on every species studied so far with regard to the axial complex, the method(s) used to infer axial complex anatomy, the specimen ID of museum specimens where applicable, and the respective references. Numbers in brackets behind "MRI" represent the resolution of the dataset. An overview of scanning parameters is provided by [24].

### Magnetic resonance imaging

Magnetic resonance imaging (MRI) was performed using the methods described by [24]. Imaging was carried out in Berlin, Germany and Würzburg, Germany using high-field small animal MRI scanners equipped with 7 T and 17.6 T super-conducting electromagnets. The resolution of the datasets varied between 20 × 18 × 18 μm^3 ^and (86 μm)^3^. Tables 1 &2 list the resolutions achieved for every species analyzed by MRI. Image processing was carried out using ImageJ 1.38w and its Volume Viewer plugin.

### Dissection

Dissection was performed on freshly fixed as well as museum specimens under direct observation through a stereo-microscope equipped with a digital camera for documentation.

### Histology

Histological analyses were performed on four echinoid species: *Eucidaris tribuloides*, *Diadema setosum*, *Psammechinus miliaris*, and *Echinocyamus pusillus*. *Eucidaris tribuloides *was collected at Twin Cayes, Belize in June 2002. *Psammechinus miliaris *and *Echinocyamus pusillus *were dredged from depths of 10–50 m in the North Sea off Helgoland, Germany in April 2005. *Diadema setosum *was purchased at a tropical fish store in Berlin, Germany in May 2005. One juvenile of *Eucidaris tribuloides*, one juvenile and three adults of *Diadema setosum*, five adults of *Psammechinus miliaris*, and three adults of *Echinocyamus pusillus *were used in this study. For light microscopy, the specimens were fixed in Bouin's fluid for 24 h, decalcified in 2% nitric acid, dehydrated in ethanol series, methylbenzoate and butanol, and embedded in paraplast (Kendall). Complete series of 8 μm thick sections were prepared using a microtome (Reichert-Jung 2050 Supercut) with steel blades (ThermoShandon Coated High Profile Disposable Blades) and later stained using an Azan staining technique [68]. Series of sections were digitally recorded with an Olympus BX 2 microscope equipped with a Color View II camera (Soft Imaging Systems).

### Electron microscopy

For transmission electron microscopy (TEM), several specimens of *Psammechinus miliaris *and *Echinocardium cordatum *were dredged from depths of 10–50 m in the North Sea off Helgoland, Germany in April 2005. The specimens were dissected and their axial complexes were fixed at 4°C in 2.5% glutaraldehyde in 0.1 M sodium cacodylate buffer at pH 7.4 for 2 h. The tissue samples were washed thrice in the same buffer, subsequently post-fixed for 1 h in 1% OsO_4 _buffered in 0.1 M sodium cacodylate, dehydrated in an acetone series and embedded in Araldite. Silver interference coloured sections (75 nm) were made with a diamond knife using an ultramicrotome (LEICA UC6), automatically stained with 2% uranyl acetate and 2% lead citrate using a semi-automatic ultrastaining machine (Phoenix), and observed with a transmission electron microscope (Philips CM 120 BioTWIN). Micrographs were made on digital imaging plates (Ditabis) and electronically processed with the software Adobe Photoshop CS3.

### Systematic classification

The systematic classification used throughout this study is based upon results obtained by [23,69-75]. Sea cucumbers (Holothuroidea) constitute the sister taxon to sea urchins, while Cidaroida are the most primitive taxon within the Echinoidea and sister taxon to Euechinoidea. The "Regularia" are a paraphyletic clade, while the Echinacea and the Irregularia each form a monophyletic taxon (Fig. 2). "Hemiasterina" and "Paleopneustina" presumably are paraphyletic clades. Resolution at the base of the Euechinoidea as well as for the Echinacea is still considered relatively poor [75].

## Results

### Definition of components of the echinoid axial complex

In its primary structure, the echinoid axial complex consists of the following components: madreporic ampulla, dorsal sac, head process, pulsating vessel, axial coelom, axial organ, canaliculi, haemal lacunae, and stone canal (Fig. 1). The designations and definitions referring to components of the echinoid axial complex as well as the developmental origin of each structure are provided in Table 3. A trilingual compilation of synonymous terms to facilitate comparative studies is provided in Table 4 with references [76-85].

**Table 3:** Definition of technical terms assigned to the axial complex of echinoids used in this manuscript.

**Structure**	**Type of body cavity involved**	**Developmental origin of body cavity**	**Topography**
Axial complex	Primary and secondary body cavities	Left larval axocoel Right larval axocoel Left larval hydrocoel Right larval somatocoel Left larval somatocoel Haemal structure	Located between madreporic plate/hydropore and ring canal, within interradius CD (sensu Lovén) below genital plate 2, lodged within the dorso-ventral mesentery, surrounded by oral and aboral somatocoel

Madreporic ampulla	Secondary body cavity	Left larval axocoel	Small space beneath the madreporic plate, connected to the exterior by madreporic pore canals, adorally gives rise to stone canal and axial coelom

Stone canal	Secondary body cavity	Left larval hydrocoel	Single tube arising from lower end of madreporic ampulla, descending towards ring canal, can be calcified, in some irregular taxa divided into three distinct parts, can be pulsatile

Dorsal sac	Secondary body cavity	Right larval axocoel	Closed cavity enclosing the head process, with muscularized epithelium, lateral to madreporic ampulla

Head process	Primary body cavity & connective tissue matrix	Haemal structure	Aboral extension of the axial organ, surrounded by the dorsal sac, pulsatile, perforated by canaliculi and haemal lacunae

Axial coelom	Secondary body cavity	Left larval axocoel	Orally oriented blindly-ending part of the axocoel, partly enwraps stone canal as well as axial organ, connects adapically to madreporic ampulla, podocyte lining

Axial organ	Primary body cavity & connective tissue matrix	Haemal structure	Parallel to axial coelom, partially surrounded by somatocoel, connected to dorso-ventral mesentery, crossed by numerous canaliculi and haemal lacunae

Pulsating vessel	Primary body cavity	Haemal structure	(Pulsatile) haemal structure running along the outside of the axial organ, protruding into the axial coelom, can be lined by muscularized epithelium, adoral extension of head process

Canaliculus	Secondary body cavity	Left larval axocoel Right larval somatococel Left larval somatocoel	Randomly distributed small invagination formed through infolding of coelomic epithelium, ending blindly, found inside the matrix of axial organ and head process

Haemal lacuna	Primary body cavity	Haemal structure	Randomly distributed anastomosing compartments within axial organ, head process and mesenteries, not lined by epithelium, part of the haemal system

A primary body cavity is always lined by extracellular matrix, whereas a secondary body cavity is lined by a mesothel (i.e. mesodermally derived epithelium). Axocoel = protocoel, hydrocoel = mesocoel, somatocoel = metacoel [4].

**Table 4:** Trilingual list of technical terms used in publications dealing with the axial complex.

**English**	**French**	**German**
**Axial complex** Axial gland complex [57] Axial hemal complex Madreporite-axial-complex	Complexe axial	Axialer Organkomplex Axial-Hydrocoel-Komplex [20] Axialkomplex [7] Axo-Hydrocoel-Komplex [61]

**Madreporic plate** Calcareous disc [76] Madrepore Madrepore plate Madreporic body Madreporic tubercle Madreporite [77] Madreporitic body Madreporitic plate Madreporite plate Sieve plate	Plaque madréporique Madrépore Madréporite	Madreporenplatte [44] Madreporit Siebplatte

**Madreporic pore canal** Hydropore Labyrinthine canal Madreporic canal Madreporic duct Madreporite opening Madreporite pore canal	Canalicule du madréporite Canal madréporique Pore madréporique	Hydroporus Kanal der Madreporenplatte Labyrinthkanal Madreporenkanal Porenkanal Porenkanälchen [16] Wimpernkanal

**Madreporic ampulla** Ampulla Ampulla of madreporite Ampulla of the stone canal Madreporic chamber Madreporic vesicle Madreporitic ampulla Sub-madreporite ampulla	Ampoule Ampoule collectrice Ampoule madréporique Ampoule sous-madréporique Canal excréteure Canal ramifié aquifére Espace infundibuliforme [46] Orifice de communication	Ampulla Ampulle [16] Madreporenblase Madreporit Protocoelampulle Sammelblase [26]

**Stone canal** Calcareous tube [76] Madreporic canal Madreporic tube Sand-canal Water canal Water tube	Canal du sable Canal hydrophore Canal madréporique Canal onduleux Canal pierreux Cœur acquifére Tube acquifére Tube hydrophore [78]	Axiales Wassergefäβ Axialwassergefäβ Steincanal [44] Steinkanal Wassergefäßkanal Wasserkanal

**Dorsal sac** Axocoel Dorsal ampulla Heart Heart lumen Madreporic vesicle Pericardial cavity Pericardium Terminal coelom Terminal sinus	Espace sous-madréporique [17] Expansion terminale du sinus axial Hydrocoele droit Rudiment de l'hydrocoele droit Sinus terminal Vésicule madréporique [45]	Axocoel Dorsalblase Dorsalsack [38] Fortsatzcoelom [26] Fortsatzsinus Hohlraum unterhalb des Madreporiten Rechter Axialsinus Sinus dextra Terminalsack

**Head process **[79] Aboral diverticulum Aboral part Aboral terminus [39] Dorsal organ Head piece Heart coelom Heart lumen Internal ridge Pericardium Terminal process	Diverticule aboral [45] Organe dorsal Processus glandulaire [45] Prolongement de l'organe axial Prolongement terminal	Aboraler Fortsatz [26] Drüsenfortsatz Eiförmiger Anhang [80] Fortsatz Fortsatz des Achsenorgans Fortsatz des Axialorgans Nebenniere [31] Terminalfortsatz

**Axial coelom** Axial canal Axial gland lumen Axial sinus Axocoel Central cavity Central lumen Lumen of axial gland	Canal excréteur de la glande Canal glandulaire Canal ovoïde Cavité de la glande brune Cavité de l'organe ovoïde Cœlome axial [66] Cœlome glandulaire [81] Cœur Organe d'excrétion Sac fusiforme [82] Sinus axial Sinus glandulaire	Axialcoelom Axialer Hohlraum Axialkanal Axialsinus Axocoel Drüsenhohlraum Höhle des sichelförmigen Bandes [44] Hohlraum Hohlraum der Niere Hohlraum des Dorsalorgans Linker Axialsinus Problematischer Kanal Schlauchförmiger Kanal [6] Sinus sinistra Ureter [31]

**Axial organ** Axial gland Axial haemal duct Axial haemal vessel Brown gland Central plexus Dorsal organ Fusiform body Genital stolon Glandular organ Kidney Oral part Ovoid gland Plexiform gland [84] Mysterious gland Septal gland Spongy body Tubular axial organ	Canal glandulaire Cavité de l'organe ovoïde Cœur [40] Corps plastidogène [78] Corps pyriforme [82] Glande brune [82] Glande excrétrice Glande madréporique [15] Glande ovoïde [46] Lacune axiale Organe axial [81] Organe d'excrétion Organe plastidogène Rein lymphoïde [81]	Achsenorgan Axialdrüse Axialorgan Braune Drüse Chromatogenes Organ [83] Dorsalorgan [6] Drüse Drüsiges Organ [16] Herz Herzförmiger Kanal [44] Lymphoides Organ Niere [31] Ovoide Drüse Septalorgan Wassergefäßherz [14] Zentralplexus [85]

**Pulsating vessel** Axial blood vessel Central contractile vessel Contractile vessel Internal ridge Irregular vessel Pulsatile vessel	Vaisseau pulsatile Cordon axial	Herz Pulsierendes Gefäβ

**Canaliculus** Diverticulum	Canalicule Diverticule du sinus axial	Canaliculus Drüsenschläuche Nebenhohlräume

**Haemal lacuna** Blood lacuna Blood vessel Haemal vessel Lacunar system	Lacune hémale Système hémal	Blutlakune Epithellose Masche Hämalkanal Lakunennetz

List of terms that have been used so far in publications dealing with the echinoid (as well as echinoderm) axial complex including madreporic plate and madreporic pore canals. The terms used in this study are printed in bold. The author(s) that first established a certain term appear after it in brackets. Where no reference is provided, the creator (author) of the respective term could not be identified with certainty.

### Morphological findings

In this section, the results derived from our own analyses are combined with results derived from the literature available on the axial complex as well as its mesenterial suspension. For a given species, these different contributions may vary considerably, and Tables 1 &2 provide a compilation.

#### Cidaroida

**Figure 3:**
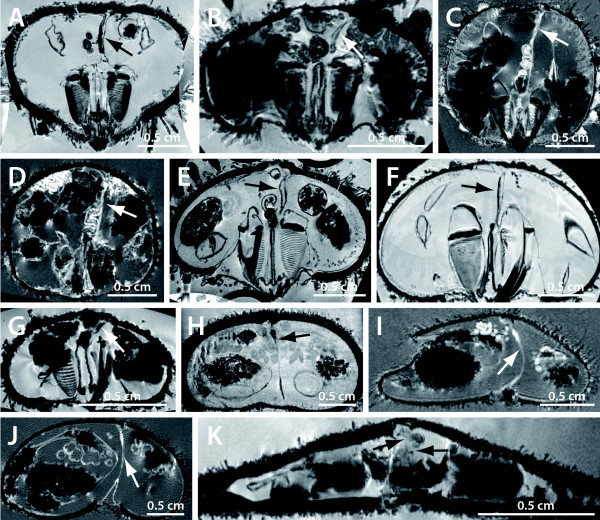
**Vertical magnetic resonance imaging (MRI) sections of various sea urchin specimens**. The virtual sections depict the axial complex (arrow). A *Cidaris cidaris *(Cidaroida). B *Caenopedina mirabilis *(Pedinoida). C *Plesiodiadema indicum *(Aspidodiadematidae). D *Salenocidaris hastigera *(Salenioida). E *Strongylocentrotus purpuratus *(Echinoida). F *Lytechinus variegatus *(Echinoida). G *Genocidaris maculata *(Temnopleuroida). H *Echinoneus cyclostomus *(Holectypoida). I *Cassidulus caribearum *("Cassiduloida"). J *Echinolampas depressa *("Cassiduloida"). K *Echinarachnius parma *(Clypeasteroida). Tables 1 & 2 list resolutions for each MRI dataset.

**Figure 4:**
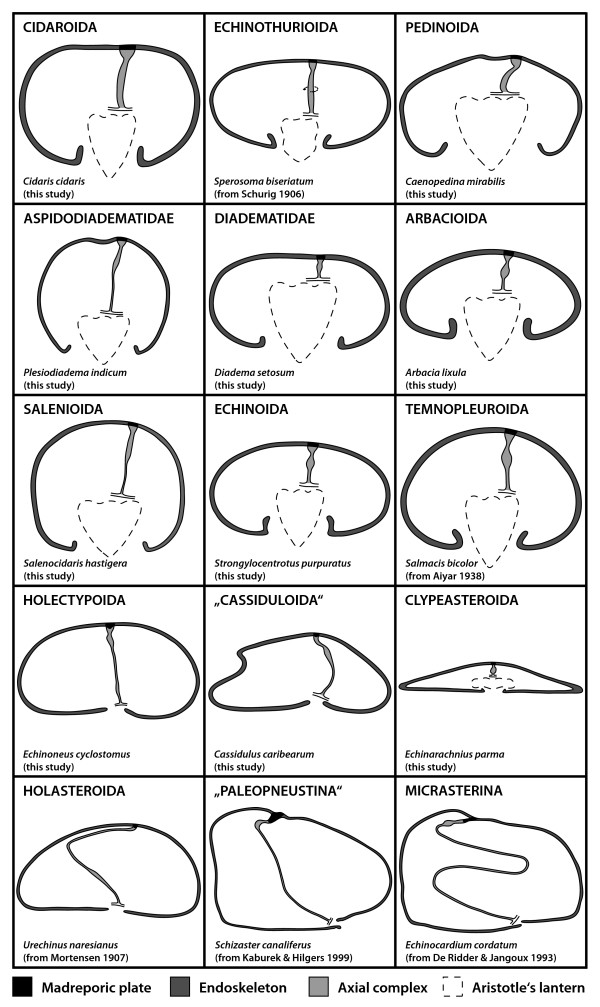
**Schematic representation of the axial complex in selected echinoid taxa**. The drawings concentrate on the gross morphology of the axial complex. All other internal organs are omitted, the ring canal is depicted in part only, and the anus is not shown. The legend indicates every structure shown.

**Figure 5:**
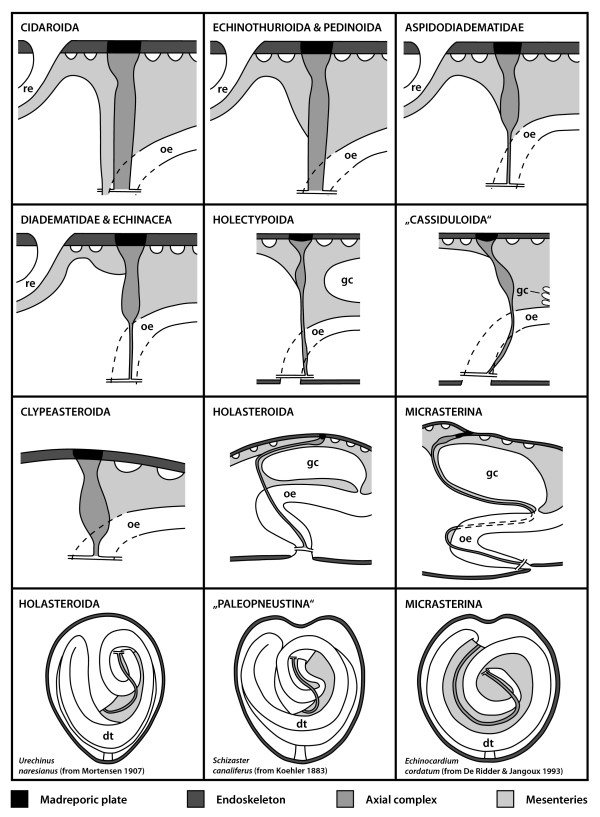
**Schematic representation of the mesenterial suspension of the axial complex in various higher sea urchin taxa**. The drawings demonstrate the impact of the gastric caecum on the architecture of the axial complex. Upper three lines: dorso-ventral mesentery always depicted on the right-hand side. Dashed lines indicate course of the oesophagus or the axial complex (Micrasterina). Lower line: Ventral view of the digestive tract and the axial complex in the vicinity of the oesophagus of highly derived irregular sea urchin taxa (Atelostomata) – note the extension of the mesentery. The grey-scale legend denominates every structure shown. dt = digestive tract, gc = gastric caecum, oe = oesophagus, re = rectum.

**Figure 6:**
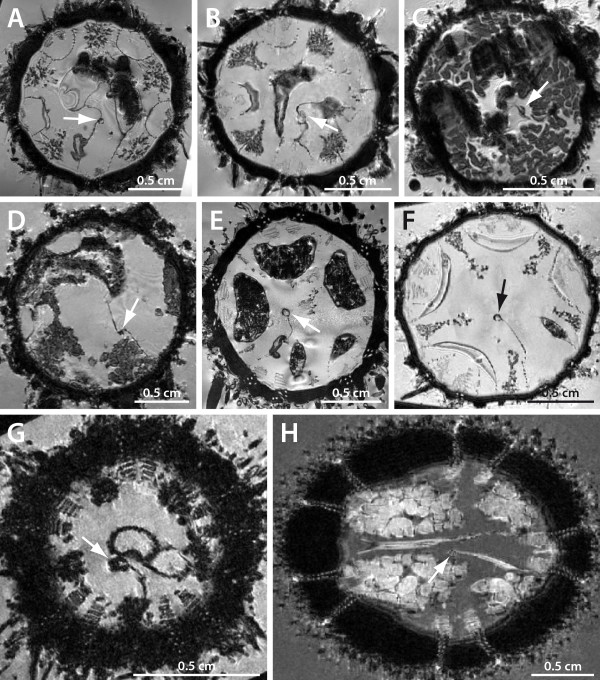
**Horizontal magnetic resonance imaging (MRI) sections of various sea urchin specimens**. The virtual sections were made at the level of gonads, upper digestive tract, and axial complex (arrow). Note the differing mesenterial suspensions of the axial complex. A *Cidaris cidaris *(Cidaroida). B *Eucidaris metularia *(Cidaroida). C *Caenopedina mirabilis *(Pedinoida). D *Aspidodiadema hawaiiense *(Aspidodiadematidae). E *Strongylocentrotus purpuratus *(Echinoida). F *Lytechinus variegatus *(Echinoida). G *Mespilia globulus *(Temnopleuroida). H *Echinoneus cyclostomus *(Holectypoida). Tables 1 & 2 list resolutions for each MRI dataset.

**Figure 7:**
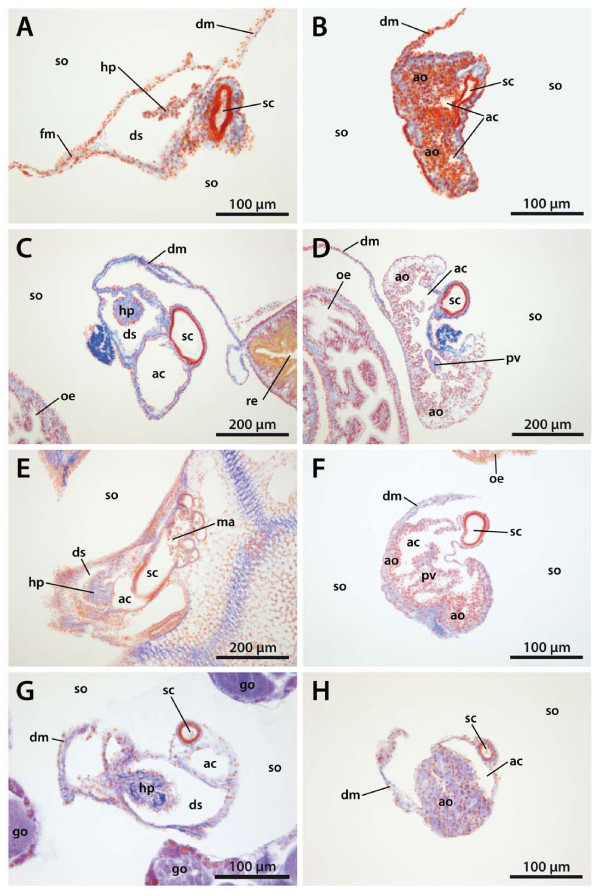
**Horizontal light-microscopic sections through the echinoid axial complex**. A, B *Eucidaris tribuloides *(Cidaroida); C, D *Diadema setosum *(Diadematidae); E, F *Psammechinus miliaris *(Echinoida); G, H *Echinocyamus pusillus *(Clypeasteroida). Left column: section at the level of head process and dorsal sac. Right column: section at the level of axial organ and axial coelom. ac = axial coelom, ao = axial organ, dm = dorso-ventral mesentery, ds = dorsal sac, fm = free mesentery, go = gonad, hp = head process, ma = madreporic ampulla, oe = oesophagus, pv = pulsating vessel, re = rectum, sc = stone canal, so = somatocoel.

In lateral view, the axial complex found in *Cidaris cidaris *and *Eucidaris metularia *is almost straight and it lies directly underneath the madreporic plate (Figs. 3A, 4). Throughout its entire course it maintains more or less the same width, only slightly bulging in the middle. It is suspended by the dorso-ventral and the free mesentery (Fig. 5). The dorso-ventral mesentery is strongly developed and connects axial complex, oesophagus, and the peripharyngeal (or lantern) coelom with the endoskeleton. The free mesentery connects the peripharyngeal coelom with axial complex and rectum. The axial complex is thus attached to two mesenteries over its entire length in *Cidaris cidaris *and *Eucidaris metularia *(Fig. 6A, B). In the juvenile specimen of *Eucidaris tribuloides*, however, the free mesentery is lacking at the level of the axial organ (Fig. 7B).

A madreporic plate is present in cidaroid species and numerous madreporic pore canals connect the exterior with the madreporic ampulla underneath the madreporic plate. Below the madreporic ampulla lies the roundish-elongated dorsal sac (Fig. 7A). Its epithelium is only slightly muscularized and mainly glandular according to [17] and [26]. However, the presence of glands at this location seems unlikely due to the enclosed nature of the dorsal sac.

**Figure 8:**
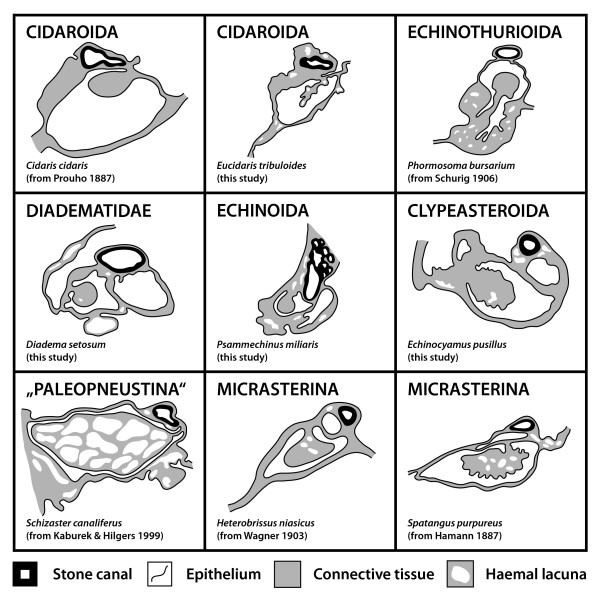
**Comparative morphology of the echinoid axial complex at the level of head process and dorsal sac**. Schematic representation of the axial complex at the level of head process and dorsal sac based on light-microscopic sections. Note that although changes in shape do occur, the internal composition remains largely the same. For better comparison, the stone canal is shown towards the top of each image. The legend indicates every structure shown.

**Figure 9:**
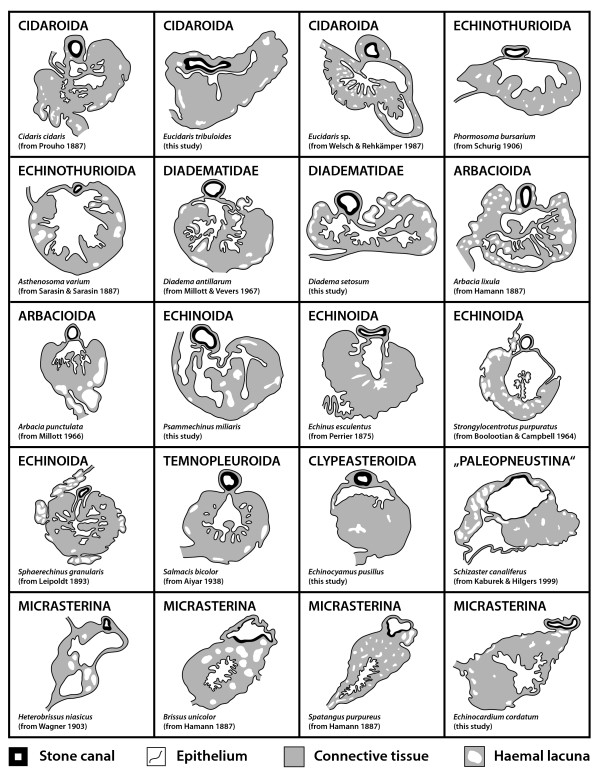
**Comparative morphology of the echinoid axial complex at the level of axial organ and axial coelom**. Schematic representation of the axial complex at the level of axial organ and axial coelom based on light-microscopic sections. Note that although changes in shape do occur, the internal composition remains largely the same. For better comparison, the stone canal is depicted towards the top of each image. The legend indicates every structure shown.

The head process, located within the dorsal sac (Fig. 8), is lined by myoepithelial cells. It extends adorally into the pulsating vessel of the axial organ. The madreporic ampulla opens adorally into the axial coelom and the stone canal. The axial coelom is a blindly-ending cavity that is located between stone canal and axial organ (Fig. 9) and that extends towards Aristotle's lantern. The width of the axial coelom does not vary much, remaining large towards the adoral end in *Cidaris cidaris *[17,26]. In *Eucidaris tribuloides*, the axial coelom in general is reduced to a thin cavity (Fig. 7B). It is lined by an epithelium with numerous podocytes. These podocytes are found to be restricted to that side of the axial coelom in *Eucidaris *sp. that borders the axial organ [27].

In all cidaroid species analyzed to date, the axial organ is a haemal structure composed of a connective tissue matrix filled with coelomocytes and anastomosing haemal lacunae. Canaliculi extend into its interior from the lining somatocoelomic and axocoelomic epithelia. The axial organ sends out a well-developed haemal lacuna towards the perioesophageal haemal ring that is in close contact with the ring canal. The stone canal is a tubular structure connecting the madreporic ampulla with the ring canal. At its adoral end, the stone canal forms several dilatations that converge with the ring canal. Ultrastructural investigations of the stone canal of *Eucidaris *sp. reveal a prismatic epithelium with ciliated, myoepithelial, and granulated cells that probably represent secretory neurons [28].

#### Echinothurioida

The axial complex of echinothurioids is straight in lateral view, located directly underneath the madreporic plate. Schurig [30] and Sarasin & Sarasin [31] report it to be spirally winding in *Phormosoma bursarium*, *Asthenosoma varium*, *Hygrosoma hoplacantha*, and *Sperosoma biseriatum *(Fig. 4). It is suspended by two mesenteries (Fig. 5), of which the dorso-ventral mesentery is strongly developed, connecting the axial complex, the oesophagus, and the peripharyngeal coelom with the endoskeleton. The free mesentery connects the axial complex with the rectum and terminates halfway down the axial complex in *Phormosoma bursarium *and *Asthenosoma varium *[30,31] (Fig. 5).

A madreporic ampulla lies underneath the madreporic plate and opens into the axial coelom and the stone canal. Sarasin & Sarasin [31] report the madreporic ampulla of *Asthenosoma varium *to be connected by a canal to another small cavity lying underneath the madreporic plate. However, these structures were not reported for *Phormosoma bursarium *[30], and we believe this isolated observation to be an artefact that will not be further considered in this analysis. The dorsal sac (Fig. 8) lies underneath the madreporic plate and is lined by a slightly muscularized epithelium that is mainly glandular [30,31]. However, in analogy to cidaroids, the presence of glands at this location seems unlikely.

The head process is lined by a muscularized epithelium as well and continues adorally in the form of the also muscularized pulsating vessel [30]. The axial coelom parallels stone canal and axial organ (Fig. 9) and extends from the madreporic ampulla towards the ring canal where it ends blindly. Its width at the base is comparable to its middle part, at least in *Asthenosoma varium *and *Phormosoma bursarium*.

The axial organ is a structure composed of a haemal tissue mesh crossed by anastomosing haemal lacunae and canaliculi. As in Cidaroida, the stone canal forms several dilatations at its junction with the ring canal.

#### Pedinoida

In lateral view, the axial complex found in *Caenopedina mirabilis *is extending straight from the lantern adapically and then obliquely in its apical part (Figs. 3B, 4). While the upper part broadens, the lower part is of the same size as the middle part of the axial complex. It is suspended by two mesenteries (Fig. 6C), the dorso-ventral and the free mesentery. The former is strongly developed and connects axial complex, oesophagus, and the peripharyngeal coelom with the endoskeleton, while the free mesentery connects the axial complex with the rectum and terminates halfway down the axial complex, thus resembling the situation found in Echinothurioida (Fig. 5). A madreporic plate is present and numerous madreporic pore canals connect the exterior with the madreporic ampulla underneath the madreporic plate.

#### Aspidodiadematidae

The axial complex of *Aspidodiadema hawaiiense *and *Plesiodiadema indicum *is mostly straight when viewed laterally and only slightly oblique in its upper half (Figs. 3C, 4). On the level of the first curvature of the oesophagus, the axial complex tapers out adorally towards the ring canal. The axial complex is attached to the dorso-ventral and the free mesentery (Fig. 6D). The dorso-ventral mesentery is strongly developed and connects axial complex, oesophagus, and the peripharyngeal coelom with the endoskeleton. The free mesentery connects the axial complex with the rectum and terminates halfway down the axial complex (Fig. 5). In its lower half, it appears that the axial complex consists only of the stone canal and the haemal lacuna(e) that connect(s) the axial organ and the perioesophageal haemal ring. The axial organ itself seems to be restricted to the upper half of the axial complex. It is spindle-shaped and bulges in its middle part (Figs. 3C, 4).

#### Diadematidae

In lateral view, the axial complex found in *Diadema setosum *is extending straight down from the madreporic plate to the ring canal. The relatively large Aristotle's lantern found in this species (as well as in most other diadematids) squeezes the axial complex between lantern and the apical endoskeleton (Fig. 4). The axial complex is suspended by the dorso-ventral and the free mesentery. The former is strongly developed and connects axial complex, oesophagus, and the peripharyngeal coelom with the endoskeleton, while the free mesentery connects the rectum with the axial complex in its uppermost part close to dorsal sac and head process. It is not present in the middle and lower parts of the axial complex (Fig. 5). In the juvenile specimen of *Diadema setosum*, the dorso-ventral mesentery interconnects peripharyngeal coelom, oesophagus, axial complex as well as the rectum (Fig. 7C, D).

The madreporic plate is perforated by numerous madreporic pore canals that connect the exterior with the madreporic ampulla. Right underneath the madreporic ampulla lies the dorsal sac that encloses the compact and roundish-elongated head process. The dorsal sac extends further adorally, paralleling axial coelom and stone canal in its lower part (Figs. 7C, 8). The head process is more prominent in its adapical part and is lined by a strong myoepithelium. It sends out the pulsating vessel into the lumen of the axial coelom in *Diadema setosum *and *Diadema antillarum*. The axial coelom is a large cavity located between axial organ and stone canal (Fig. 8). It ends blindly towards the adoral end of the axial complex.

The axial organ bulges in the middle part of the axial complex and shows considerable infolding into the axial coelom (Fig. 7D). The stone canal is a tubular structure that constantly decreases in diameter on its way down towards the ring canal. It joins the ring canal trough a number of small canals.

#### Arbacioida

In lateral view, the axial complex is straight, extending down from the madreporic plate to the ring canal [33,34] (Fig. 4). In the vicinity of the oesophagus, the adoral part of the axial complex tapers out and reaches the surface of Aristotle's lantern. Its mesenterial suspension resembles the diadematid/echinacean type (Fig. 5). The madreporic plate is perforated by numerous madreporic pore canals. A pulsating vessel constitutes the adoral extension of the head process. Schematic representations of horizontal sections of the axial organ, axial coelom as well as stone canal of *Arbacia lixula *and *Arbacia punctulata *illustrate the internal morphology of the arbacioid axial complex (Fig. 9). The axial organ of *Arbacia lixula *is reported to be surrounded by a large number of haemal lacunae [35]. The axial complex found in *Stomopneustes variolaris *(*incerta sedis*, presumably sister taxon to Arbacioida) resembles the arbacioid/echinoid gross morphology.

#### Salenioida

The axial complex found in *Salenocidaris hastigera *(Figs. 3D, 4) is comparable in its lateral aspect to the axial complex found in aspidodiadematids. The mesenterial suspension resembles the diadematid/echinacean type (Fig. 5).

#### Echinoida

The axial complex found in the Echinoida is straight in lateral view, situated slightly shifted from the oral-aboral axis underneath the madreporic plate (Figs. 3; 4; 6E, F). At the junction with the oesophagus, the axial complex gradually tapers out towards the ring canal. Above this junction, a conspicuous swelling is visible that is widest in its middle part, giving the axial complex a spindle-shaped appearance. The mesenterial suspension resembles the diadematid/echinacean type (Figs. 5; 6E, F).

The madreporic plate is perforated by numerous madreporic pore canals that are lined by a ciliated epithelium and that merge adorally to form the madreporic ampulla (Fig. 8). The latter is lined by a ciliated epithelium as well. The diameter of the madreporic ampulla becomes gradually smaller towards its adoral end and it basally diverges into stone canal and axial coelom. The dorsal sac lies laterally underneath the madreporic ampulla and surrounds the globular head process (Fig. 8). The dorsal sac is a closed cavity and is not connected to either madreporic ampulla, stone canal or the axial coelom. Boolootian & Campbell [56], however, report a divergent morphology of the dorsal sac in *Strongylocentrotus purpuratus *in which the dorsal sac communicates with the somatocoel via a small slit and is divided into two contractile chambers. We believe this isolated finding to be an artefact and will not consider it any further in our analysis.

A strong pulsating vessel is present in all echinoid species studied so far, its ultrastructure resembling that of the head process. The axial coelom is located in-between stone canal and axial organ (Fig. 9), extending adorally before ending blindly above the ring canal. The axial organ of Echinoida is spindle-shaped in vertical section and shows characteristic infolding, with an enlarged surface towards the axial coelom with numerous digitations that protrude into the lumen of the latter.

The stone canal is located at the lateral edge of the axial complex (Fig. 9). After its gradual opening at the lower end of the madreporic ampulla, it runs down towards the ring canal, while exhibiting a diameter that varies only slightly and a shape that is almost circular in cross-section. At the lower end of the axial complex, the haemal lacuna(e) of the axial organ adorally merge(s) with the perioesophageal haemal ring while the stone canal directly opens into the ring canal. In numerous echinoid species of Echinoida the stone canal was found to pulsate.

##### Echinoida: ultrastructural findings

**Figure 10:**
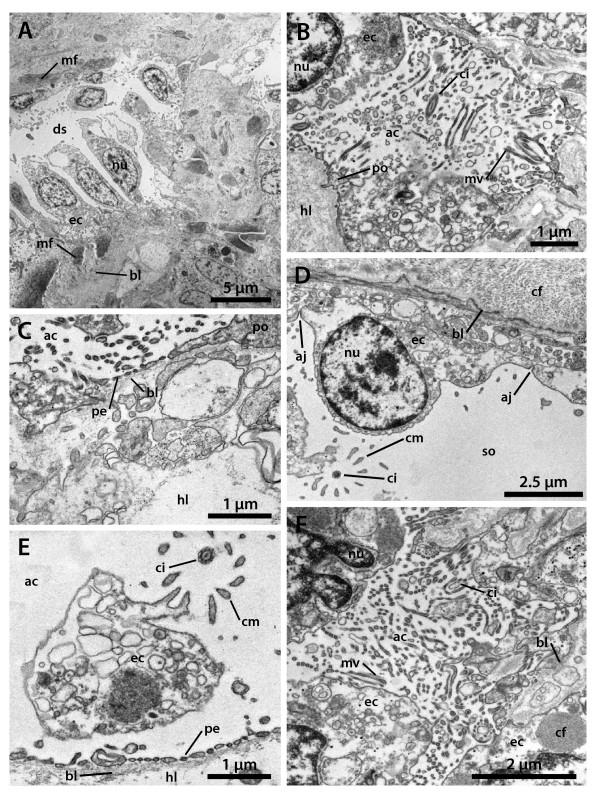
**Horizontal electron-microscopic sections of the echinoid axial complex**. A-D *Psammechinus miliaris *(Echinoida) and E, F *Echinocardium cordatum *(Micrasterina). A Dorsal sac epithelium with myoepithelial cells. B Heavily ciliated canaliculus inside the axial organ. C Axial coelom epithelium with podocyte and haemal lacunae. D Somatocoelomic epithelium. E Axial coelom epithelium with podocyte and haemal lacunae. F Heavily ciliated canaliculus inside the axial organ. ac = axial coelom, aj = adhaerens junctions, bl = basal lamina, cf = collagenous fibers, ci = cilium, cm = circumciliary microvillum, ds = dorsal sac, ec = epithelial cell, hl = haemal lacuna, mf = myofibrils, mv = microvillum, nu = nucleus, pe = pedicel, po = podocyte, so = somatocoel.

**Figure 11:**
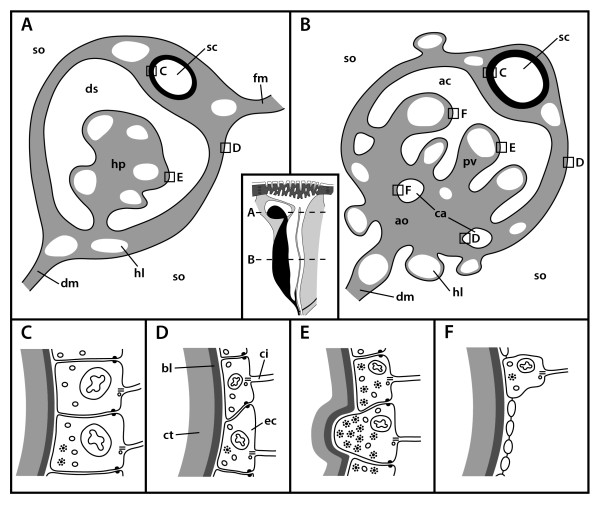
**Schematic representation of sections through the axial complex of *Sphaerechinus granularis *(Echinoida)**. A Horizontal section through the echinoid axial complex at the level of head process and dorsal sac. B Horizontal section through the echinoid axial complex at the level of the axial organ. The insert between A and B (compare Fig. 1) roughly outlines where the virtual sections were made. C Schematic representation of the columnar epithelium of the stone canal. D Schematic representation of the flat epithelium of the somatocoel. E Schematic representation of the myoepithelium that lines head process and pulsating vessel. F Schematic representation of the specialized epithelium with podocytes found in the axial coelom. ac = axial coelom, ao = axial organ, bl = basal lamina, ca = canaliculus, ci = cilium, ct = connective tissue, dm = dorso-ventral mesentery, ds = dorsal sac, ec = epithelial cell, fm = free mesentery, hl = haemal lacuna, hp = head process, pv = pulsating vessel, sc = stone canal, so = somatocoel.

In *Psammechinus miliaris*, a ciliated, pseudostratified monolayer lines the dorsal sac (Fig. 10A). While only some of the peripheral lining cells are epithelio-muscle cells, those resting on the matrix of the head process form a strong myoepithelium (Fig. 11E). Here, the epithelio-muscle cells contain strong, basally located bundles of myofilaments that form a regular network of rectangularly arranged outer circular and inner longitudinal bundles that are embedded in the voluminous matrix of the head process. The perikarya deeply extend into the dorsal sac lumen, which indicates that the organ was apparently fixed during contraction. The state of contraction can be estimated from the position of the apical adhaerens junctions between adjacent cells. Intraepithelial nerve fibre processes were found interspersed among the lining cells, but podocytes were never found.

The head process contains primary body cavities lined by the matrix of the head process. Numerous cells with homogenous cytoplasma and relatively big nuclei float inside this cavity, and some haematocytes could be recognized by their large number of lysosomes and residual bodies.

The axial organ begins at the level of the first curvature of the oesophagus and is surrounded by numerous haemal lacunae (Fig. 11B). That part of the axial organ which bulges into the axial coelom contains numerous deep crypts and invaginations in *Psammechinus miliaris*, so that the axocoelomic surface of the axial organ is tremendously enlarged. Inside the canaliculi, the entire lumen seems to be occupied by microvilli (Fig. 10B). Only a few cells are found inside the lumen, most of them coelomocytes. Haemotocytes at different stages of differentiation are present within the matrix. They contain residual bodies and lysosomes of different rank.

Numerous podocytes rest on the axial coelomic side of the matrix (Figs. 10C, 11F). Their pedicels are bridged by electron-dense diaphragmata. Each podocyte has a single cilium with a (9 × 2)+2 axoneme that adheres to the cell body by a basal body, short rootlets and an accessory centriole perpendicular to the basal body. A circle of 9–12 strong microvilli surrounds each cilium (Fig. 10D). Additional microvilli emanate from the surface of the perikaryon and only seldom from the pedicels. In *Echinometra *sp., the podocytes within the axial coelom lining are irregularly distributed and not restricted to the side covering the axial organ [27].

The stone canal lining in *Psammechinus miliaris *is a monolayered columnar epithelium (Fig. 11C). Each of these epithelial cells bears a long cilium with a (9 × 2)+2 axomene that adheres to the cell body with a basal body and three rootlet structures. A circle of 9–12 strong microvilli surrounds each cilium and additional smaller microvilli emanate from the cell surface. A number of glandular cells that show a distinct polarity can be found within the epithelium: some cells of the stone canal lining contain large, electron-densely stained vesicles. These possible secretory granules lie near the apex while other cellular components are located more basally. However, degrading cells can rarely be found among the basal portions of the lining cells, and epithelio-muscle cells, muscle cells or podocytes were never seen to be part of the stone canal epithelium. The somatocoel is lined by a flat epithelium which is only slightly muscularized (Figs. 10D, 11D). The cilia of the somatocoel epithelium show the characteristic composition with a dense microvilli fringe surrounding the axoneme. The epithelial cells are connected to each other via adhaerens junctions and do not show the specializations of the dorsal sac and axial coelom lining.

#### Temnopleuroida

In lateral view, the axial complex found in *Mespilia globulus *and *Salmacis bicolor *is straight, extending down from the madreporic plate to the ring canal (Fig. 4) [65]. In the vicinity of the oesophagus, the adoral part of the axial complex tapers out and reaches the apical surface of Aristotle's lantern. The mesenterial suspension of the axial complex resembles the diadematid/echinacean type (Figs. 5, 6G). A schematic representation of a horizontal section through the axial complex of *Salmacis bicolor *at the level of the axial organ is shown in Fig. 8. In *Genocidaris maculata*, the course of the axial complex is slightly oblique (Fig. 3G).

#### Irregularia

Most obviously, irregular sea urchin species are distinguished from animals belonging to "regular" orders by their secondarily developed bilateral symmetry. However, their analysis demonstrates that the shape of the axial complex is primarily not dependent on features of the endoskeleton, but is influenced by the architecture of several internal organs as detailed below.

#### Holectypoida

The axial complex found in *Echinoneus cyclostomus *extends from the madreporic plate down to the ring canal in an almost straight line (Figs. 3H, 4). The madreporic plate can be seen bulging slightly into the interior of the animal. In the upper fourth of the axial complex, a conspicuous swelling can be seen, which is interpreted as the axial organ [22]. Close to the ring canal, the lower fourth of the axial complex shows a swollen region which has been seen with MRI but has also been described by [22]. Unfortunately, histological data are currently not available for this taxon.

The mesenterial suspension of the axial complex resembles the aspidodiadematid type (Fig. 5). The dorso-ventral mesentery extends between oesophagus, axial complex, gastric caecum, pentagonal apical membrane (or aboral sinus), and the endoskeleton (Figs. 5, 6H). At the apical pole this mesentery joins the pentagonal apical membrane which forms the connection between the gonoducts and the axial complex. This membrane appears to be present in all irregular sea urchin taxa [8]. A second mesentery suspends the axial complex towards the rectum and also forms part of the pentagonal apical membrane. The gastric caecum is not in close contact with the axial complex (Fig. 5).

#### "Cassiduloida"

The axial complex found in *Cassidulus caribearum *and *Echinolampas depressa *extends from the madreporic plate down to the ring canal. It is bent anteriorly towards ambulacrum III (Figs. 3I, J; 4). Jensen [23] has reported similar findings for *Apatopygus recens*. The madreporic plate can be seen bulging only slightly into the interior of the animal. In the upper third, a conspicuous swelling of the axial complex can be seen which is interpreted as the axial organ. During its entire path, the axial complex is attached to the dorso-ventral mesentery. This structure extends between oesophagus, axial complex, gastric caecum, pentagonal apical membrane and the endoskeleton (Fig. 5). A second, smaller mesentery suspends the axial complex towards rectum and aboral endoskeleton as well as the pentagonal apical membrane. The gastric caecum of *Cassidulus caribearum *and *Echinolampas depressa *is reduced to numerous smaller sacs that are located further anteriorly [24] and that are therefore not in close contact with the axial complex. In *Cassidulus caribearum*, a slightly swollen region can be seen in the adoral part of the axial complex (Fig. 3I). Unfortunately, histological data are currently not available for this taxon.

#### Clypeasteroida

Due to the flattened aspect of clypeasteroids (sea biscuits and sand dollars), the axial complex is considerably miniaturized. It is located underneath the madreporic plate and has a straight (Fig. 4) to bean-shaped form in lateral view. The madreporic plate is perforated by numerous madreporic pore canals that connect the madreporic ampulla to the exterior. In *Echinocyamus pusillus *(and all other taxa of the Fibulariidae), the madreporic ampulla is in contact with the exterior via a single canal, the hydropore (*sensu *Mortensen [86]). Except for a small lateral process, the madreporic ampulla is almost tubular so that hydropore and madreporic ampulla seem to be merely an aboral elongation of stone canal and axial coelom in this species. All other clypeasteroids studied so far possess a genuine madreporic plate and a prominent madreporic ampulla, and their axial complex is attached to a single mesentery during its entire course, the dorso-ventral mesentery (Fig. 5). In its upper part, it fuses with the pentagonal membrane at the apical pole. According to Cuénot [66], clypeasteroid species possess a strongly developed dorsal sac that surrounds the muscularized head process (Figs. 7G, 8). Furthermore, Cuénot [48] reports that the dorsal sac of *Echinodiscus bisperforatus *communicates with the somatocoel via a small slit. Such a connection could not be found in *Echinocyamus pusillus*. At least in the latter species, a pulsating vessel could not be detected (Figs. 7H, 9).

The axial coelom originates from the madreporic ampulla directly underneath the madreporic plate. The axial organ can be found to be straight (*Echinocyamus pusillus*) or bean-shaped (*Echinarachnius parma, Mellita quinquesperforata*) in lateral view (Fig. 3K). The stone canal's diameter continuously decreases before passing into the ring canal in *Echinocyamus pusillus*. In the few clypeasteroid taxa that possess a gastric caecum, this highly reduced structure is not in close contact with the axial complex.

#### Holasteroida

In lateral view, the axial complex found in Holasteroida is C-shaped and extends between madreporic plate and ring canal (Fig. 4). In its upper half, the axial complex runs parallel to the aboral endoskeleton towards interambulacrum 5. The madreporic plate is slightly infolded. The conspicuous swelling right underneath the madreporic plate, interpreted as the axial organ, is in an horizontal position. In its middle section, the previously horizontally oriented axial complex bends towards the mouth, comes into close contact with the oesophagus adorally, and leads in a straight line down towards the ring canal. Another swelling can be observed in its lower third, at least in *Urechinus naresianus *(Fig. 4). Unfortunately, no histological data are currently available for this taxon.

The axial complex is held in place by the dorso-ventral mesentery that attaches digestive tract, axial complex, gastric caecum, and pentagonal membrane to the calcite endoskeleton. Between gastric caecum and axial complex, this mesentery is very thin, so that both structures are in close contact with each other in the aboral part of the animals. A second, smaller mesentery connects the axial complex with rectum, pentagonal apical membrane and endoskeleton. The mesenterial strand that interconnects the first loop of the digestive tract is extending half-ways along the gut loop (Fig. 5). Mortensen [21] describes the gross morphology of the axial complex in four holasteroid species: *Echinosigra paradoxa*, *Pourtalesia jeffreysi*, *Pourtalesia wandeli*, and *Urechinus naresianus*. The axial complexes found in *Antrechinus nordenskjoldi *and *Pourtalesia hispida *closely resemble this description. The large gastric caecum is in close contact with the upper part of the axial complex (Fig. 5).

#### "Hemiasterina"

The axial complex found in *Hemiaster expergitus *closely resembles the holasteroid and paleopneustine axial complex in its gross morphology.

#### "Paleopneustina"

In lateral view, the axial complex found in *Abatus cavernosus *and *Schizaster canaliferus *is C- to L-shaped and extends down from the madreporic plate to the ring canal close to the mouth (Fig. 4). The following description is based on *Schizaster canaliferus *[20]. The madreporic plate is characterized by two calcareous ridges protruding into the interior of the endoskeleton. The axial complex is attached to the right ridge from where it is extending towards interambulacrum 5. It then bends down towards the mouth and extends along the gastric caecum and later the oesophagus before finally reaching the ring canal. It is primarily suspended by the dorso-ventral mesentery that attaches digestive tract, axial complex, gastric caecum, and pentagonal apical membrane to the calcite endoskeleton. Between gastric caecum and axial complex this mesentery is very thin, so that both structures are in close contact in the aboral part of the specimen (Fig. 5). A second, smaller mesentery attaches the axial complex to rectum, pentagonal apical membrane and endoskeleton.

The madreporic ampulla is reduced to several broadened madreporic pore canals inside the right calcareous ridge, and according to Kaburek & Hilgers [20], the dorsal sac as well as a pulsating vessel are not present in *Schizaster canaliferus*. However, the structure of the head process resembles that in the "regular" species (Fig. 8). The axial organ is located directly behind the right calcareous ridge and is oriented horizontally, parallel to the endoskeleton. A large uniform axial coelom is not present. The stone canal is tri-partite: its upper part bears a tall-prismatic epithelium, its middle region is muscularized and filled with coelomocytes, while its lower part close to the ring canal is lined by a flat epithelium and is only slightly filled with coelomocytes. The stone canal of *Schizaster canaliferus *is not dilated at its junction with the ring canal and at least its muscularized middle part can pulsate. The mesenterial strand that interconnects the first loops of the digestive tract is extending half-ways along the first gut loop (Fig. 5, bottom line). The large gastric caecum is in close contact with the upper part of the axial complex (Fig. 5).

#### Micrasterina

A lateral view reveals that the micrasterine axial complex is roughly S-shaped. Its gross morphology is largely similar in the micrasterine taxa studied so far (Table 2). The madreporic plate protrudes into the interior by two calcareous ridges comparable to the paleopneustine species *Schizaster canaliferus*. Lodged in-between these two ridges and successively emanating from the right ridge, the axial complex is oriented horizontally in its first small part. During its later course, it is closely associated with the large gastric caecum passing along anteriorly towards ambulacrum III. At the junction of the gastric caecum with the oesophagus, the axial complex bends backwards towards interambulacrum 5 before finally moving again anteriorly towards the ring canal that encircles the mouth (Figs. 4, 5). The micrasterine axial complex is primarily suspended by the dorso-ventral mesentery that attaches digestive tract, axial complex, gastric caecum, and pentagonal membrane to the calcite endoskeleton (Fig. 5). Between gastric caecum and axial complex, this mesentery is very thin, so that both structures are in close contact in the aboral part of the specimens. A second, smaller mesentery suspends the axial complex towards pentagonal apical membrane, rectum, and aboral endoskeleton. The mesenterial strand that interconnects the first loops of the digestive tract is extending far towards ambulacrum III, "dragging" the axial complex along (Fig. 5).

The madreporic plate is perforated by a small number of horizontal and vertical madreporic pore canals [16,18]. Underneath and within the right calcareous ridge, a small madreporic ampulla can be seen. Parallel to this structure, a dorsal sac can be found at least in *Echinocardium mediterraneum*, *Heterobrissus niasicus*, and *Spatangus purpureus *[16-18]. It contains the head process (Fig. 8), which is not pulsatile in *Heterobrissus niasicus *according to Wagner [18]. The head process extends further posteriorly into the axial organ. A pulsating vessel was found in several micrasterine species. Parallel to axial organ and stone canal, the axial coelom extends adorally from the inconspicuous madreporic ampulla.

The gross morphology of the stone canal is known for a number of micrasterine taxa and closely resembles the architecture in *Schizaster canaliferus*. It can be sub-divided into three parts: the first part is in close association with the axial organ and ends at the adoral end of the latter. Here, the stone canal transforms into a canal composed of a mesh-like tissue that is filled with "pigment" [16]. It continues in this state parallel to the gastric caecum and the haemal lacunae until reaching the vicinity of the oesophagus where it transforms again to become a hollow canal covered by a thin epithelium. Schurig [30] states that the water vascular and the haemal systems in *Heterobrissus niasicus *are always clearly separated, the haemal lacuna running parallel to the stone canal before reaching the perioesophageal haemal ring.

##### Micrasterina: ultrastructural findings

In *Echinocardium cordatum*, the axial coelom is lined by flat epithelial cells, each of which bears a single cilium. The ciliary axoneme shows the typical (9 × 2)+2-pattern and is attached to the basal body. Two ciliary rootlets and a basal foot anchor the cilium to the cell. Both ciliary rootlets are co-axial, running obliquely to the main axis of the basal body. Some fibrillar material secures the connection of the accessory centriole to the longer vertical rootlet and fixes its position underneath the basal foot. The cilia are encircled by a dense fringe of 9–12 strong microvilli (Fig. 10E). Numerous podocytes line the axial coelom and the lumina of the canaliculi inside the axial organ (Fig. 11F). The canaliculi are filled with a huge number of microvilli and cilia that project into their lumen, resulting in a tremendous surface extension (Fig. 10F). Epithelio-muscle cells can be detected sporadically, and sometimes even podocytes seem to bear muscle fibers.

The axial organ consists of a dense matrix and is rich in canaliculi and lacunae (Fig. 9). It is characterized by cells with electron-bright cytoplasm and by the distinct lacunar system. Local increments of the matrix constitute haemal lacunae filled with haemal fluid. A basal lamina demarcates the border of the tissue towards the coelomic epithelium of the axial coelom. Connective tissue cells and an enormous number of phagocytotic cells can be found in the inner parts of the axial organ. The haematocytes are filled with residual bodies as well as lysosomes of different rank. Collagenous and elastic fibers as well as free cells constitute the major part of the axial organ. The somatocoelomic lining is a flat, only slightly muscularized epithelium (Fig. 11D), consisting of a monociliated monolayer overlying the basal lamina of the axial organ. No podocytes could be detected in the somatocoelomic epithelium.

## Discussion

Whereas an extensive comparative study of the axial complex had been undertaken in the sister taxon to Echinoidea, the Holothuroidea [11], such a comparative approach has not yet been applied to the study of the echinoid axial complex. One objective of this study was therefore to obtain an insight into the overall anatomy of the axial complex within a wide selection of sea urchin species. Many of these species can be obtained only with great difficulty as viable specimens, and several others are available only as fixed specimens from museum collections, often precluding their use for dissection, histology or electron microscopy. To partially circumvent these problems, we additionally employed a non-invasive imaging technique (MRI) in conjunction with a thorough review of the relevant literature dating back to the pioneering studies of F. Tiedemann that were published at the beginning of the 19^th ^century [44]. This complementary approach did not allow an analysis of all examined echinoid species with all procedures, but nevertheless permitted to gather results from a representative number of sea urchin species regarding the architecture of the axial complex, thereby forming the basis for an in-depth comparison. To avoid, as much as possible, repetitions with data presented in the Results section, we will attempt to concentrate here on (i) major changes in axial complex architecture, (ii) the possible reasons for these changes, and (iii) the axial complex' potential function as derived from morphological features. Finally, (iv) we address the question whether phylogenetically informative characters can be deduced from comparative observations of the axial complex. It must be borne in mind, however, that the present compilation is far from complete and rests, in a number of orders, on the analysis of only a few or even single species and specimens.

### Major changes in the architecture of the axial complex and its constituent parts

The gross morphology of the axial complex in the analyzed sea urchin taxa varies considerably, although major structural changes are restricted to the more derived irregular species (Fig. 4). However, the form of the axial complex may depend on a number of factors, such as the age or health of the specimen. Furthermore, since the stone canal fuses with the ring canal set atop Aristotle's lantern in the "regular" and clypeasteroid taxa, movement of this feeding apparatus may alter the form of the axial complex considerably. In the Atelostomata, the axial complex is running parallel to the endoskeleton in its upper part before bending down adorally to run along the gastric caecum towards oesophagus and ring canal. This can be considered the most derived condition. Other irregular sea urchin taxa such as the Holectypoida, "Cassiduloida" and Clypeasteroida have obviously preserved the more plesiomorphic state, exhibiting an axial complex that is comparable in its gross morphology to the condition observed in Aspidodiadematidae or Diadematidae, the "regular" taxa presumably most closely associated with the ancestor of Irregularia (Fig. 2).

In most species analyzed here, the madreporic plate (Figs. 1, 4) is situated medially on the aboral side and is perforated by numerous madreporic pore canals. However, *Echinocyamus pusillus *does not have a madreporic plate, and its axocoel is connected to the exterior via a single pore (or sometimes several pores). Mortensen [86] describes the reduction of the madreporic pore canals as characteristic for the Fibulariidae, a small group within the Clypeasteroida that exhibit strong paedomorphic traits, and the reduction of madreporic pore canals has been considered a highly derived feature within the Clypeasteroida [87].

All examined "regular" species possess a prominent madreporic ampulla (Figs. 1, 4), while the madreporic ampulla is reduced to a system of broadened madreporic pore canals in the spatangoid sea urchins as shown by [20] for *Schizaster canaliferus*. Whether the ampulla-like cavity underneath the madreporic plate that was described by Koehler [15] and Hamann [16] in *Spatangus purpureus *can be referred to as a madreporic ampulla must currently be regarded as unclear.

A prominent, in cross-section C-shaped dorsal sac (Figs. 1, 4, 11) is partially enveloping the head process in most species. According to [20], this structure is reduced in *Schizaster canaliferus*. Hamann [16] and Wagner [18], however, presented drawings of the dorsal sac and head process in *Echinocardium mediterraneum *and *Heterobrissus niasicus*, which, together with the comparison of the axial complex architecture in several spatangoid taxa presented here, leads us to assume that a misapprehension of its composition may have led Kaburek & Hilgers [20] to consider that the dorsal sac is missing in *Schizaster canaliferus*. However, we hypothesize that size and form of dorsal sac and head process may be dependent on the sexual maturity of the specimen, since, at least in Asteroidea, the dorsal sac-head process complex was shown to serve the gonads as a local heart [88].

The axial coelom of all species studied ends blindly at the adoral end of the axial organ (Figs. 1, 11). The axial coelom, like dorsal sac and madreporic ampulla, shows a similar structural pattern within the "regular" sea urchin species as well as the clypeasteroid species examined so far, although it must be noted that species of Atelostomata appear again to display a differing condition (see, e.g. [20]).

Rather unclear is the situation regarding the pulsating vessel (Fig. 9). Cidaroid, echinothurioid, diadematoid, arbacioid, echinoid, as well as temnopleuroid species have been reported to possess a truly pulsating structure within the axial coelom that constitutes an adoral extension of the muscularized head process. Such pulsations have not been reported for any irregular taxon, although histological sections of the upper axial coelom of spatangoid species reveal a separate haemal lacuna overlying the axial organ that we homologize with the pulsating vessel found in the "regular" taxa. Whether this structure actually pulsates cannot currently be stated with confidence.

The axial organ is present in all echinoid species observed so far and constitutes an integral component of the sea urchin haemal system both in "regular" and irregular taxa (Figs. 1, 7, 11). Its internal structure is comparable in all taxa, its dense connective tissue matrix being crossed by numerous anastomosing haemal lacunae and canaliculi.

The stone canal (Figs. 1, 7, 8, 9, 11) is in fact that sub-structure of the axial complex undergoing the most drastic changes during evolution: in spatangoid species it is divided into three distinctive parts, its upper part closely resembling the stone canal found in "regular" taxa. At the adoral end of the axial organ it transforms to become a chambered structure filled with numerous coelomocytes. This part of the stone canal is additionally surrounded by smooth muscle fibers and might therefore pulsate. The lower part is again unchambered, but is also filled with coelomocytes. Kaburek & Hilgers [20] believe that the stone canal in *Schizaster canaliferus *may assume functions of the axial organ. This, however, would imply a mixing of different body fluids at this location which we believe to be highly unlikely.

The columnar epithelium of the stone canal is a distinctive feature in comparison to the flat epithelia of axial coelom and somatocoel (Fig. 11). The interior of the stone canal is filled with numerous cilia that cause an active and directed transport of coelomic fluid [28,54,59]. Another difference to the other coelomic cavities can be seen in the presence of glandular cells, which Rehkämper & Welsch [28] consider to be neuro-secretory cells. Neither muscular tissue or podocytes within nor contractions by the stone canal could be observed by us in "regular" or irregular sea urchins.

In conclusion, it can be stated that presumably all sea urchin taxa possess an axial complex as part of their haemal system. The axial complexes found in all "regular" species are comparable to a large degree, consisting of a straight to slightly oblique structure located in interradius CD extending vertically down from the madreporic plate to the ring canal. In the irregular sea urchin species an evolutionary trend towards miniaturization and horizontal alignment of the axial organ in conjunction with a bending of the axial complex as a whole (Fig. 4) can be observed. However, despite these drastic changes in axial complex gross morphology, its internal composition has largely remained unchanged, so that the plesiomorphic Cidaroida share numerous similarities with the highly derived Atelostomata.

### Reasons underlying the observed changes in axial complex architecture

The mesenterial suspension of the axial complex and of the digestive tract components is straightforward in the "regular" taxa: Cidaroida possess an axial complex supported by two mesenteries, of which the free mesentery is successively reduced in the other "regular" taxa (Fig. 5). The situation observed in the juvenile specimen of *Eucidaris tribuloides*, where only the dorso-ventral mesentery is suspending the axial complex in its middle part (Fig. 7B), might hint at a similar development within Cidaroida or might be due to the early developmental stage of the specimen. In *Psammechinus miliaris*, both mesenteries seem to be reduced completely in the lower middle and lower part of the axial complex [45, this study], hinting at an even further reduction of mesenterial structures in some taxa within the Echinoida. The mesenteric suspension of the axial complex in the more primitive Irregularia can be described as largely comparable to the condition observed in Aspidodiadematidae and Diadematidae. Whether the smaller mesentery suspending the axial complex towards the rectum can be considered homologous with the respective structure in "regular" sea urchins cannot currently be answered with certainty. Notable is the absence of this mesentery in the Clypeasteroida, a condition that presumably can be ascribed to the laterally displaced position of the rectum. In the Atelostomata, the dorso-ventral mesentery fuses with the large mesenterial strand that interconnects the coils of the first loop of the digestive tract (Fig. 5). However, the precise mesenterial suspension of irregular sea urchins on the whole as well as the homologies of the respective mesenterial structures remain unclear and merit further investigation.

The assumption that a reorganization of the morphology of the axial complex occurred along with the evolution of bilateral symmetry can therefore be ruled out. Rather, the present results suggest a gradual co-evolution of various internal organs within the Irregularia based on their structural interdependence. The reasons underlying this development can be seen in the altered lifestyles of irregular sea urchins based on their conquering of infaunal habitats and a parallel change in feeding strategies. These changes apparently correspond to the gradual enlargement of the gastric caecum, a structure that constitutes an outgrowth of the proximal stomach, which is thought to be of importance in the digestive process [19]. However, due to its topography, the gastric caecum is suspended by the dorso-ventral mesentery as well. Its successive growth inevitably had to lead to a rearrangement of the gross morphology of the axial complex. Since the gastric caecum is most prominent in the Atelostomata, its effect on the structure of the axial complex and the dorso-ventral mesentery should also be most evident here, which is precisely what is observed (Figs. 4, 5). An additional change in digestive tract anatomy is responsible for another highly derived condition seen in Micrasterina: here, the mesentery suspending the first coil of the digestive tract has expanded and moved in anterior direction towards ambulacrum III, "dragging" the axial complex along, and ultimately leading to the more or less S-shaped form observed in this group of animals (Figs. 4, 5).

Another aspect of importance for the changes in axial complex gross morphology can be seen in the relative position of the apical system of the endoskeleton that accommodates also the madreporic plate. In the Holasteroida, the apical system is located further anteriorly than in other irregular taxa, which, in combination with the posteriorly orientated gastric caecum, brings about a prolonged and horizontally aligned axial complex in the apical region (Figs. 4, 5).

### Function of the echinoid axial complex

In terms of function, the sea urchin axial complex remains as enigmatic as ever, especially so, since these marine invertebrates have been shown to survive its removal for several months [36,49,89], although some specimens do show rapid regeneration of the removed structures [36], suggestive of a beneficial function of this structure for the animal. Historically, a number of functions have been attributed to the echinoid axial complex, including that of a gill [90], an immune organ (e.g. [35,37,91,92]), a gland (e.g. [7,15,46,57,93]), an embryonal organ without function in the adult [94], a coelomocyte and cell production site (e.g. [16,17,66,89,95]), a cell degradation site (e.g. [45,60]), a swelling of the water vascular system [14], and a heart (e.g. [40,44,55,56,96-99]). Furthermore, a number of authors have advocated a predominantly excretory role of the axial complex (e.g. [16,17,31,46,100,101]). This hypothesis is supported by recent studies that homologize the left larval axocoel (protocoel) that can be found throughout the "lower" deuterostome taxa (e.g. [13,102,103]). In this context, the finding of podocytes – highly specialized cells that permit selective fluid transfer – in coelomic compartments of the axial complex is indicative of an excretory function [27].

One aim of our study concerned the possible presence of podocytes in the Irregularia to support the excretion hypothesis as well as to shed light on other functions that have been ascribed to the echinoid axial complex. Since epithelio-muscle cells are found lining the axial coelom in addition to podocytes, their contraction could generate a pressure gradient that allows filtration of the haemal fluid into the axocoel, but it remains unsolved on the basis of the available data whether the ultrafiltrate constitutes primary urine in terms of a metanephridial system [104]. However, the presence of structures as highly specialized as podocytes leads us to assume that the axial organ at the junction of haemal and coelomic compartments primarily acts as an excretory structure.

The epithelium of the dorsal sac consists of a strong pseudostratified myoepithelium in the vicinity of the head process in *Psammechinus miliaris*. At least in the "regular" sea urchins ultrastructurally examined so far (Table 1), this can be stated as the common situation. The presence of axons inside the dorsal sac lining of asteroids [105] clearly supports the assumption that the myoepithelial lining of the dorsal sac is a contractile component of the axial complex. Based on the "local heart-hypothesis" postulated for Asteroida by Warnau & Jangoux [88], we hypothesize that the echinoid dorsal sac may serve to spatially separate the contractions of the head process from the neighbouring body cavities. The fact that no podocytes were found lining the dorsal sac is in support of its complete disconnection from other coelomic cavities and the exterior.

The inner composition of the head process with its free cells and elastic fibers resembles that of the axial organ, allowing for the haemal fluid to pass unhindered. The distinct muscularization of the surrounding epithelium as well as the even macroscopically visible contractions in some species (e.g. [35,47,49,55,61] corroborate the axial organ's function as a local contractile organ. Since the haemal spaces inside the head process are directly connected to the gonadal haemal lacunae, the head process might serve to supply the aborally located gonads with nutrients. If so, enlargement of the digestive tract and reduction of the number of gonads should be accompanied by the reduction of haemal supply lacunae. This can in fact be observed in Spatangoida [19]. The remarkable amplification of the axial organ surface by involution (Fig. 11) clearly supports the assumption that substances in solution are being absorbed by large areas of the axial organ. This situation leads us to assume that components of all body fluids (haemal fluid and coelomic fluid) might interact inside the axial organ. In addition, the large number of haematocytes in the axial organ points to a removal of corpuscular wastes, such as foreign cells (e.g. bacteria) and cellular remnants of the animal itself. As the genomic analysis of *Strongylocentrotus purpuratus *has revealed the presence of many immune system-related genes [106], it would clearly be interesting whether these loci exhibit a particularly pronounced expression within components of the axial complex.

### Phylogenetic implications

Based on the comparative morphological results, a number of characters can be inferred that are phylogenetically informative. However, since we believe all axial complex sub-structures (Table 3) to be present in all echinoid orders, no informative characters can be deduced from the presence or absence of these.

The overall form of the axial complex shape in lateral view differs considerably in sea urchin taxa and ranges from a straight aspect to a largely S-shaped form in the derived Micrasterina. A further character that can be used with confidence is the orientation of the axial organ, being vertically oriented in most taxa and horizontally oriented in the Atelostomata only. The mesenteric suspension of the axial complex differs among the observed taxa and clear-cut changes are observed between "regular" and irregular taxa. In both groups a successive reduction of the free mesentery can be observed (Fig. 5), in the Clypeasteroida this suspension is entirely absent. The mesenteric suspension in Micrasterina further distinguishes this taxon from other Atelostomata with its enlarged mesenteric strand interconnecting the coils of the first gut loop.

A character for which extensive data are available is the composition of the stone canal. In the Atelostomata, the stone canal is tri-partite whereas all "regular" taxa observed so far possess a uniform stone canal. However, the conspicuous swelling observed in the oral part of the stone canal in *Echinoneus cyclostomus *and some "Cassiduloida" might hint at a sub-divided stone canal in these taxa as well. Here, more extensive histological examination is required. A further character involving the stone canal is its connection to the ring canal which is dilated in the basal echinoid taxa. Echinacea have presumably developed a single connection. However, the condition is not known in several basal "regular" (e.g. Pedinoida, Aspidodiadematidae) and irregular taxa (e.g. Holectypoida, "Cassiduloida") as well as in the outgroup, Holothuroidea. Another character for which no sufficient data are available is the interesting aspect of the diverging distributional pattern of podocytes in Cidaroida and Echinoida [27]. To shed light on this phenomenon, extensive ultrastructural analyses are required.

In order to assess whether the above mentioned characters will lead to an extension of the morphological character matrix and thus to a refined echinoid phylogeny, a cladistic analysis based on these and further soft tissue characters is currently in preparation by members of our group. It remains to be determined whether the comparative analysis of further echinoid organs, such as the gut and its various appendages, will result in the recognition of additional features whose architecture is dependent on each other within the Echinoidea.

## Conclusion

Our findings reveal a structural interdependence of various internal organs, including digestive tract, mesenteries, and the axial complex within the Echinoidea. The present study further demonstrates that the type of approach employed here, i.e. to combine all available data on a given organ across a large number of taxa, long in use for hard-part anatomical structures, can be applied to internal organs as well and is very powerful in elucidating interdependent anatomical relationships that are not obvious when the analysis is carried out only with a few species.

## List of abbreviations

CAS: Califonia Academy of Sciences, San Francisco; MNHN: Muséum Nationale de la Histoire Naturelle, Paris; MRI: magnetic resonance imaging; NHM: Natural History Museum, London; ULB: Université Libre de Bruxelles; USNM: National Museum of Natural History, Washington, D.C.; ZMB: Systematische Zoologie am Museum für Naturkunde, Berlin; ZMH: Zoologisches Institut und Museum, Hamburg; ZSM: Zoologische Staatssammlung, München.

## Competing interests

The authors declare that they have no competing interests.

## Authors' contributions

AZ designed and coordinated the study, carried out the dissections and histological analyses, prepared specimens, carried out scanning, and wrote the manuscript. CF prepared and scanned specimens. TB carried out TEM observations and supervised the experiments. All authors read, edited, and approved of the final manuscript.
